# Bupropion Causes Misdiagnosis in Brain Dopamine Transporter Imaging for Parkinsonism

**DOI:** 10.1097/WNF.0000000000000359

**Published:** 2019-07-29

**Authors:** Emma A. Honkanen, Nina Kemppainen, Tommi Noponen, Marko Seppänen, Juho Joutsa, Valtteri Kaasinen

**Affiliations:** ∗ Department of Neurology, University of Turku; † Division of Clinical Neurosciences, Turku University Hospital; ‡ Department of Clinical Physiology and Nuclear Medicine, Turku University Hospital, University of Turku; § Turku Brain and Mind Center, University of Turku, Turku, Finland.

**Keywords:** bupropion, SPECT, parkinsonism, depression

## Abstract

The objective of this study was to report long-lasting effects of bupropion on brain dopamine transporter (DAT) in a patient with depression and parkinsonism.

**Methods:**

The patient was a 52-year old man who had been treated with 150 mg/d of bupropion for depression. The patient developed cognitive problems, bradykinesia, and reduced stride length for which he was scanned with [^123^I]FP-CIT single photon emission computed tomography after the recommended 1-week discontinuation of bupropion. Levodopa treatment trial was initiated without a response. Eleven months later, the patient was scanned for a second time after a 1-month stoppage of bupropion.

**Results:**

The first scan was abnormal with left putamen specific binding ratio of 1.99 (SDs from the reference value mean, −2.40), right putamen of 2.27 (SD, −1.84), left caudate of 2.33 (SD, −2.26), and right caudate of 2.29 (SD, −2.18). The second scan (after 1-month discontinuation) was normal, and specific binding ratios had increased from 5.2% to 31.7% in all striatal regions as compared with the first scan. Brain magnetic resonance imaging and [^18^F]fluorodeoxyglucose positron emission tomography imaging were normal, and there was no levodopa response or other features supporting neurodegenerative parkinsonism.

**Conclusions:**

Bupropion has previously generally been discontinued 1 week prior DAT imaging, which meets the recommended, albeit arbitrary, time interval of 5 plasma clearance half-lives before the scan. One-week discontinuation of bupropion before DAT imaging may be insufficiently short. Our case shows that longer medication washout and rescan may be needed when there is contradiction between the imaging result and clinical outcome in patients with medications affecting DAT binding.

Striatal dopamine transporter (DAT) imaging can be used in the differentiation of degenerative parkinsonian disorders from conditions without such degeneration.^1,2^ Early to moderate Parkinson disease (PD) patients typically have a clear 50% to 60% loss in putaminal DAT binding compared with healthy individuals.^3^ However, several drugs that affect dopaminergic neurotransmission can influence the imaging outcome, thus biasing the interpretation. According to the prescription information for [^123^I]FP-CIT provided by the US Food and Drug Administration and the European Medicines Agency, amphetamine, benztropine, cocaine, methylphenidate, phentermine, sertraline, and several other drugs can induce changes in tracer binding.^4,5^ One of these drugs is bupropion, a norepinephrine-dopamine reuptake inhibitor, which is widely used as an antidepressant or as an aid to smoking cessation.^6^ Because of its dopaminergic mechanisms of action, bupropion has also shown some efficacy for dopamine agonist-mediated compulsive behaviors^7^ and apathy^8^ in PD.

Potentially interfering drugs should generally be stopped at least 5 plasma clearance half-lives before the scan to avoid effects on [^123^I]FP-CIT binding.^9^ Bupropion has an elimination half-life of approximately 20 hours,^6^ and according to the current recommendations, a 1-week washout should be sufficient for an accurate analysis of striatal DAT binding.

Here, we present a patient who had an abnormal result regarding [^123^I]FP-CIT single photon emission computed tomography (SPECT) after a 1-week discontinuation of bupropion, and the scan results later returned to normal after a considerably longer break in medication use.

## CASE REPORT

A 52-year-old man with a medical history of atrial fibrillation had suffered from depression for several years. A psychiatrist referred the patient to a neurologist owing to problems in cognition, balance, and verbal communication. His antidepressant medications consisted of 225 mg/d of venlafaxine and 150 mg/d of bupropion. The patient was receiving no antiparkinsonian medication or other drugs known to have effects on the dopamine system. Family history was unremarkable.

A clinical examination showed mild bradykinesia in the left hand, mild slowness in his foot-tapping rate, and reduced stride length. Neuropsychological tests demonstrated some loss in memory functions and a lack of concentration. Blood and urine laboratory tests were unremarkable.

**FIGURE 1:**
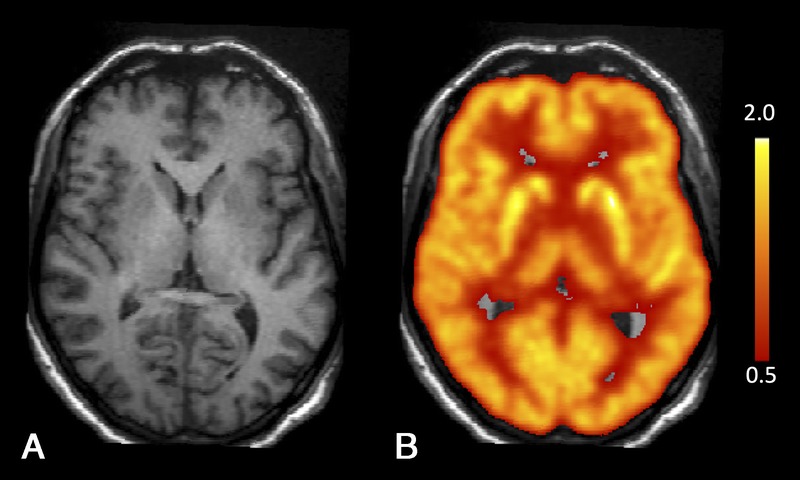
A, T1-weighted 1.5 T brain magnetic resonance imaging showing no abnormal findings. B, Normal brain glucose metabolism on [^18^F]FDG positron emission tomography. The figure shows [^18^F]FDG uptake normalized to the average uptake of the whole brain (red-yellow scale).

**FIGURE 2:**
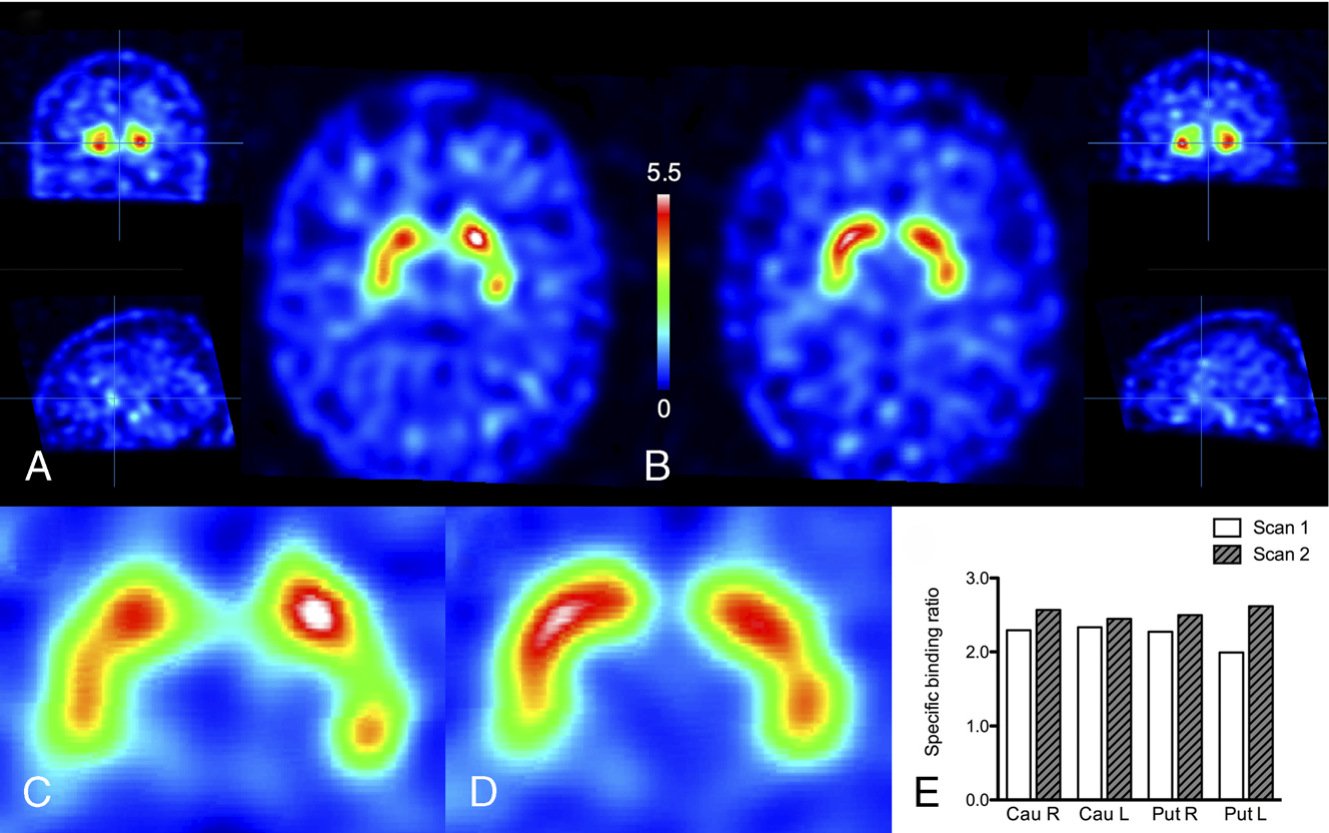
A and C. First [^123^I]FP-CIT scan after 1 week of bupropion washout. B and D. Second scan with corresponding slices after a 4-week washout. Color scale indicates [^123^I]FP-CIT binding relative to the occipital cortex binding (specific binding ratio [SBR] +1). E. Regional [^123^I]FP-CIT SBR values.

Brain magnetic resonance imaging showed no abnormalities, and the [^18^F]fluorodeoxyglucose positron emission tomography imaging was normal (Fig. 1). Because of partially asymmetric bradykinesia, [^123^I]FP-CIT SPECT was performed 7 days after discontinuation of bupropion use. Dopamine transporter imaging showed reduced binding bilaterally but particularly in the left putamen (left putamen specific binding ratio [SBR] of 1.99 [standard deviations from the reference value mean (SD), −2.40], right putamen SBR of 2.27 [SD, −1.84], left caudate SBR of 2.33 [SD, −2.26], and right caudate SBR of 2.29 [SD, −2.18]) (Fig. 2A, C).

After the first scan, a levodopa treatment trial with 450 mg/d was initiated without a clear response. Because of the lack of a levodopa response and the history of bupropion use, SPECT imaging was repeated 11 months after the first scan. The patient was now instructed to discontinue bupropion 4 weeks before the scanning. Bupropion (150 mg/d) was replaced by agomelatine (25 mg/d) owing to the long discontinuation. According to the Montgomery-Asberg Depression Rating Scale, the level of depression was similar between the 2 SPECT scans (37/60 and 39/60 at the time of the first and second scan, respectively), and the patient confirmed that the discontinuation of bupropion had been carried out as instructed before both scans.

The follow-up scan was analyzed using identical methods as those used for the first scan. The imaging results now showed quantitative overall improvement of the SBR values to a normal range (left putamen SBR of 2.62 [SD, −1.09], right putamen SBR of 2.50 [SD, −1.37], left caudate SBR of 2.45 [SD, −2.01], and right caudate SBR of 2.57 [SD, −1.62]) (Fig. 2B, D). The second scan was also evaluated visually as normal. There was a 31.7% increase in the [^123^I]FP-CIT SBRs in the left putamen and 5.2% to 12.2% increase in other regions (Fig. 2E). Importantly, nonspecific background binding stayed constant between studies (first study, 117.26 counts/voxel; second study, 117.48 counts/voxel). All brain images were coregistered together using a mutual information algorithm implemented in the Statistical Parametric Mapping software (SPM12, http://www.fil.ion.ucl.ac.uk/spm/software/spm12/).

## DISCUSSION

Bupropion and its active metabolite, hydroxybupropion, cause a DAT occupancy of no more than 20% to 25% during treatment.^10,11^ This level of occupancy has been suspected to be too low to be the primary therapeutic mechanism of the drug.^10,11^ Nevertheless, our case demonstrated that the effect of a clinical dose of bupropion on DAT binding remains robust after a 1-week discontinuation, which may be owing to long-term adaptive mechanisms on dopamine transmission. This interferes with the [^123^I]FP-CIT SPECT results and can mislead the clinical interpretation. It has been previously reported that the use of bupropion could also interfere with the imaging results of [^99m^Tc]TRODAT-1, another tropane-derivative DAT tracer; the binding values improved after 14 days of discontinuation of the medication but, importantly, still to lower levels than the reference values.^12^ In our patient, the binding returned to completely normal after a 1-month discontinuation of bupropion.

The case further suggested that the effect of bupropion may mimic neurodegeneration, as the loss of binding was not equal in the caudate and putamen, but it was predominantly seen in the left putamen 1 week after discontinuation. However, the DAT binding pattern was not typical for PD, because the reduced binding was seen mainly in the anterior part of the left putamen, not in the posterior putamen (Fig. 2C). It is also important to note that our patient had predominant motor symptoms in the left hand and the most severe bupropion-related DAT binding defect was observed in the ipsilateral side of the striatum, not in the contralateral hemisphere as is typically the case in PD. These differences in the regional distribution of DAT binding defects may be critical in the visual interpretation of scans that are taken from patients under dopaminergically active medications, such as bupropion. If a clear binding loss is detected, but the binding pattern does not appear typical for parkinsonism or clinically relevant, a potential effect of a drug is a possibility. However, it should be noted that, although our clinical findings were in agreement with a drug effect, and the scans were analyzed using semiquantitative and visual methods, we cannot exclude the possibility of minor inconsistencies in repeated DAT scans. In addition, the later scan can be characterized a scan without evidence of dopaminergic deficit, a common finding in clinical populations of patients with suspected PD.^13^ According to present knowledge, patients with scans without evidence of dopaminergic deficit who have true neurodegenerative parkinsonism are extremely rare,^14^ and a normal presynaptic dopamine function in functional imaging is currently considered an absolute exclusion criterion for PD.^15^

The prescription information for [^123^I]FP-CIT states that bupropion may interfere with imaging, but the impact of the drug on imaging has not been fully established.^4,5^ In many imaging centers, bupropion is discontinued for 7 to 10 days before [^123^I]FP-CIT scanning, which satisfies the suggested, albeit arbitrary, recommendation of 5 half-lives before the scan.^9^ Our case suggests that 5 half-lives may not be sufficient and that the interval should probably be increased to 1 month to avoid misdiagnoses of dopaminergic defects in neurological patients.
